# Correlation of Transfusion Dependence and Its Associated Sequelae to Hematological and Biochemical Parameters in Patients With Sickle Cell Disease and Beta Thalassemia Major in Khobar: A Retrospective Study

**DOI:** 10.7759/cureus.42151

**Published:** 2023-07-19

**Authors:** Khaled S Albahout, Mohammed Yunus, Yaser G Mohammad, Adnan F Almalki, Saleh K Alduailej, Basel Z Alanazi

**Affiliations:** 1 General Surgery, Imam Abdulrahman Bin Faisal University, Dammam, SAU; 2 Pathology, Imam Abdulrahman Bin Faisal University, Dammam, SAU; 3 Medicine, Imam Abdulrahman Bin Faisal University, Dammam, SAU

**Keywords:** transfusion dependence, laboratory parameters, clinical picture, beta thalassemia major, sickle cell disease, anemia

## Abstract

Sickle cell disease (SCD) and beta thalassemia major (βTM) are multisystemic, genetically inherited diseases. They are caused by mutations of hemoglobin, which ultimately cause abnormal functioning of the red blood cells. The morbidity and mortality rates of these diseases are significant, as they may result in severe complications, some of which are quite fatal; hence, early diagnosis and treatment are crucial. The purpose of this study is to collect patients’ data in terms of their manifestations and overall clinical picture and correlate them to the laboratory parameters with emphasis on their transfusion dependence and its sequelae in King Fahd Hospital of the University (KFHU), Al-Khobar, Saudi Arabia.

After obtaining ethical approval from the institutional review board and in collaboration with the blood bank, patients' data were retrospectively collected from the hospital's database and categorized into two disease groups. Accordingly, data related to the biological and demographic information, clinical picture pattern, laboratory investigations, and therapeutic measures, with emphasis on blood transfusion as a treatment option, were gathered and analyzed. Eventually, the aforementioned data aspects were assessed for the probability of correlations, which were proven to be present to some level as an answer to our cohort study's question. Such findings, which will be depicted later in this study, might represent a ground for having a more comprehensive and extensive approach in terms of the general evaluation of patients with SCD and βTM based on the established level of correlation.

During the course of conducting our research, we encountered some limitations, including the sample size and scarce data available during the process of data collection.

## Introduction

Sickle cell disease (SCD) is a spectrum of inherited hemoglobinopathies caused by gene defects that code for beta-globin of the hemoglobin [1,2]. A single gene defect of adenine to thymine builds a substitution of hydrophobic valine instead of hydrophilic glutamic acid at position 6 of the 11th chromosome [3-6]. There are many subtypes that exist within SCD, including sickle cell anemia (SCA), which is the most common type where both alleles are defective in the homozygous condition (HbSS), sickle cell trait (HbAS), where one chromosome has the gene in the heterozygous state, hemoglobin SC disease (HbSC), and hemoglobin sickle-beta-thalassemia [1,3]. Usually, the manifestations of the disease start to occur after the age of six months as fetal hemoglobin (HbF) starts to decrease to the adult's level [3]. SCA is characterized by chronic hemolytic anemia, demanding blood transfusions, unpredictable pain crises, and widespread organ damage [1,2].

Thalassemia, on the other hand, is a group of hereditary, chronic, hemolytic, hypochromic, microcytic anemia caused by quantitative reduction or absent synthesis of α-globin chains (α-thal) or β-globin chains (β-thal) [7-9]. β thalassemia particularly occurs due to a wide range of point mutations of the β-globin gene on chromosome 11 [7]. It is divided into three main categories depending upon their clinical presentation: major, intermedia, and minor [9]. Beta thalassemia major (βTM) is brought about by a homozygous defect, characterized by a complete absence of β-globin chains, leaving behind an excess number of α chains [8]. Its diagnosis is made readily within the first two years of life, as patients present with severe anemia requiring massive blood transfusion therapy on a regular basis to keep the level of hematocrit 27-30% to suppress erythropoiesis [5,7-9]. In addition to blood transfusion, they require iron chelation therapy to prevent overload and other serious complications [10,11].

Epidemiology

The incidence of SCD differs by state, race, ethnicity, and other factors [2]. According to the Global Burden of Disease study's systematic analysis, 3.2 million individuals have SCD, and 176,000 deaths occur yearly from SCD-related complications [6]. The prevalence of SCD is more in Sub-Saharan Africa [1,2]. Regarding Saudi Arabia, the prevalence of SCD reached up to 1.4% in some regions [12]. These prevalence statistics are not based on neonatal screening and are likely to underestimate the real prevalence of SCD. Consanguineous marriage rates in this region of the globe approach 50% [12].

According to the Saudi Premarital Screening Program, SCD prevalence was estimated to be 0.26%, notably the Eastern Province having the highest prevalence of 1.2% [13]. In a local study with newborn screening for SCD in the Eastern Province, the frequency of SCD was 2.6% (compared to 1.2% from premarital screening) [12]. According to hospital-based research from the Eastern region, 73% of deaths occur in those under the age of 30 years, with acute chest syndrome (ACS) and infection being the leading causes of mortality [12].

β-thalassemias are most common in the Mediterranean region, the Middle East, and the Indian subcontinent. It is becoming more widespread in non-endemic locations as a result of migratory trends [10,14]. In 2008, the World Health Organization (WHO) reported that more than 40,000 newborns are born with β-thalassemia yearly, of whom approximately 25,500 are transfusion-dependent β-thalassemia [14]. The prevalence of β-thalassemia in the Middle East has typically been high, owing to a high carrier rate and a societal desire for consanguineous marriages. In Saudi Arabia, the prevalence of β-thalassemia when compared to neighboring countries in the Middle East is considered one of the highest (0.05%) [15].

Pathophysiology

Hemolysis and vaso-occlusive crisis (VOC) are the two main features of SCD [1]. In comparison with hemoglobin A (HbA), sickle hemoglobin (HbS) has decreased oxygen affinity [2]. In a deoxygenated condition, the HbS molecule is vulnerable to converting into stiff, elongated polymers ending with a sickle appearance [1,3]. There are factors that precipitate the sickling process such as infection, dehydration, cold, acidosis, or hypoxia [3]. Sickling in short, results in shortened erythrocyte survival and disrupted cell flow, which eventually leads to vascular occlusion and tissue infarction [3].

Regarding βTM, anemia is caused by a reduction in hemoglobin synthesis, which leads to a rise in HbF and hemoglobin A2 (HbA2), as there are reduced β-chains for HbA production [7]. Also, extra α-chains result in insoluble aggregations and produce precipitates that harm the membranes of RBCs, resulting in intra-medullary hemolysis, and this is of most pathological significance in βTM [7,8]. Severe anemia and erythroid hyperplasia, as well as bone marrow enlargement and extra-medullary hematopoiesis (EMH), result from this inefficient erythropoiesis [7]. Multiple transfusions cause chronic iron deposition in multiple organs [8].

Ineffective erythropoiesis (IE) is a process characterized by increased proliferation of RBCs. In such a condition, the bone marrow becomes expanded leading to EMH. Such a phenomenon is seen when various body tissues contribute to the formation of RBCs. With the continuum of this process, characteristic deformities occur and become obvious in the face and bony structures [16]. Although it has been well-recognized that IE is associated with thalassemia, researchers have recently found some evidence relating it to SCD [17,18].

In βTM, the pathophysiological events have been primarily linked to increased proliferation of RBCs [19]. Therefore, splenectomy might be a solution to minimize the transfusion requirement [19]. However, the risk of infection as well as cardiovascular events rise greatly if such a measure was to be considered [20]. Additionally, IE is also linked to hepcidin over-expression leading to impaired iron metabolism [16,19,21].

Clinical picture and complications

VOC is the most common cause of hospitalization and ER visits [22]. It is also referred to as a painful crisis and is experienced when multiple sickled erythrocytes adhere and get lodged in the circulation [22]. Dactylitis is often the presentation of VOC in infants and toddlers [23]. Also, avascular necrosis (AVN) of hips and shoulders can occur. Osteomyelitis cases are more common in SCD, with the most common causative organisms being *Staphylococcus aureus*, *Streptococcus pneumoniae*, and *Salmonella* [24]. Additionally, one of the most common complications in SCD patients is ACS, which is an acute lung injury syndrome, associated with new lung infiltration on chest X-ray [25]. Manifestations are usually chest pain, fever, tachypnea, wheezing, cough, or hypoxemia, which could be self-limiting episodes or sometimes they advance to acute respiratory failure leading to death or morbidity [24,25]. Fat embolism syndrome secondary to VOC in multiple bones, especially the pelvis and femur, leads to bone marrow infarction and subsequently the release of the content including fat to the bloodstream, which can lead to pulmonary embolism and is a major risk factor for ACS [25]. Another complication is priapism, which could be self-limiting or may need emergency treatment [23]. Spleen involvement is almost always in SCD, and with the absence of the protective effect of HbF, it is usually infarcted in the first 18-36 months of life [26]. Also, splenic sequestration is one of the life-threatening conditions. It is often accompanied by viral or bacterial infection [24]. Furthermore, SCD patients are at risk of stroke [23]. Acute anemia with accelerated hemolysis leads to what is called a hyper-hemolytic crisis, manifesting as an acute reduction in hemoglobin associated with a reticulocyte count higher than the baseline [24]. As for transfusion complications, they are similar to βTM. They include alloimmunization, infectious diseases such as hepatitis C virus (HCV) and hepatitis B virus (HBV), allergic reactions, hemolytic transfusion reactions, febrile reactions, and iron overload. SCD patients are relatively protected from hemosiderosis as compared to βTM patients [27].

Regardless of the efforts and advancements in βTM management, patients still suffer from a number of complications [28,29]. In relation to blood transfusion complications, the common ones are similar to those seen in SCD [28,30]. Although the incidence of new infections is less, HCV is common in βTM patients [28]. In long-term RBC transfusion, iron accumulates in body organs leading to hemosiderosis and causing multiple-organ damage [31-33]. Hepatic disease is being reported as the second cause of death in βTM. As for musculoskeletal complications, expansion of the bone marrow and reduced bone volume are seen [28]. As a consequence, osteopenia and osteoporosis develop [28]. Examples of these skeletal abnormalities are prominent frontal bossing, delayed pneumatization of the sinuses, marked overgrowth of the maxillae, malocclusion of the teeth, depressed nasal bridge, and canting of the eyes [28]. Furthermore, the shortening of the limbs, particularly the arms, is seen [32]. Lastly, pathological fractures can happen, which are not common but rather remarkable [32,33]. Other important causes are endocrinopathies, such as hypothyroidism, hypoparathyroidism, diabetes mellitus, and hypogonadism, which contribute to bone diseases [28]. Endocrinopathies are commonly seen in transfusion-dependent βTM patients, where iron burden causes various abnormalities. Effects include growth hormone deficiency, resulting in delayed growth, short stature, and infertility [32,33]. Delayed puberty and hypogonadism are mainly due to siderosis in the pituitary or gonads or both [33]. Moreover, diabetes mellitus is seen as β-cell destruction taking place due to excessive siderosis and insulin resistance [33]. Hypothyroidism in βTM is more to be seen in the second decade of life [33]. Hypersplenism is another complication. If the spleen is affected, it compromises the immune status with an association of leukopenia, thrombocytopenia, and increased requirement for transfusion; some patients may require splenectomy in such a case [33].

Diagnostic evaluation

SCD patients are mostly diagnosed through prenatal screening tests, including hemoglobin electrophoresis [4]. The electrophoresis findings in SCD SS form predominantly include HbS, some amounts of HbF, and HbA2 with no HbA, while those of the other form, SCD SC, are composed of HbS and hemoglobin C (HbC) [34]. Moreover, routine laboratory tests might be done on those patients and they include complete blood count (CBC) with differentials, renal function test (RFT) with electrolytes, liver function test (LFT), and other metabolic panel tests [4]. Blood culture or other sepsis work-up tests might be obtained depending upon the suspicions of an ongoing infectious process. Determining blood type and crossmatch might be warranted in case of a possible blood transfusion [35-38]. Besides laboratory tests, radiological imaging might be indicated in certain cases. Plain radiography (X-ray), for example, might be obtained for patients with suspected ACS. Similarly, those with suspected hepatosplenomegaly or abdominal lesions might need to get ultrasonic (US) imaging. SCD patients from the age of two years are recommended to get a trans-cranial US to assess for cerebrovascular accidents (CVAs)/strokes to prevent them. Other imaging modalities might be indicated based on clinical judgment [4]. It must be noted that early diagnosis of SCD is of paramount importance to reduce the overall risk of morbidity and mortality [2].

For βTM, usually, children are clinically diagnosed by the age of fewer than two years [7]. It is attributed to the fact that patients start to have anemia symptoms near the age of six months. However, for the diagnosis to be confirmed, hemoglobin electrophoresis is needed similar to SCD [7]. Routine tests like CBC, metabolic panel, and peripheral blood picture (PBP) might be needed [7,39].

Transfusion-dependence management

Various therapeutic strategies can be considered along the course of anemia treatment, which can range from simple analgesia to undertaking strong measures, such as regular blood transfusion and even stem cell transplantation in certain cases. Blood transfusion, which is the mainstay treatment of severe βTM and SCD, is used to improve the oxygen-carrying capacity of the blood and minimize the risk of disease complications [40,41]. If a patient’s condition necessitates receiving at least one unit of packed RBCs (PRBCs) over a specified interval, such a condition is referred to as transfusion-dependent anemia [42]. Generally, undertaking treatment measures in such pathologies depends upon various factors, including the patient’s clinical background, blood hemoglobin levels, and the severity of anemia.

As far as SCD is concerned, RBC transfusion is still the mainstay of treatment whether its complications are acute or chronic, as it is linked to reduced rates of morbidity and mortality [40]. It is estimated that around 90% of SCD adult patients will receive at least one blood transfusion along their illness course [43]. The use of transfusion is increasing as indications are generally expanding and the use of iron chelators is accompanied by it since iron overload post-transfusion can be quite reduced [44,45]. When it comes to indications of transfusion, different conditions may entail transfusion and they include CVAs, transient ischemic attack (TIA), ACS, multi-organ failure, acute decrease in hemoglobin levels without reticulocytosis, hepatic/splenic sequestration, priapism, acute exacerbation of anemia, and severe sepsis [40,45]. It is recommended that βTM patients should receive PRBC unit(s) with a 40 g hemoglobin content when transfusion is indicated [46]. The blood content must be leukoreduced to prevent adverse reactions, as WBCs may result in fatal complications if they enter the recipient’s circulation [47].

Pharmacological treatment

In addition to blood transfusion, there are several medications approved and used for certain indications in either type of anemia. It is to be noted that bone marrow transplant is the only option for cure whereas all the other options act as temporary solutions [48]. Such medicines include hydroxyurea to reduce the risk of painful crises, episodes of ACS, blood transfusions, and rate of hospitalization, and penicillin, which reduces the risk of pneumococcal bacterial infections in patients with previous history or those who underwent splenectomy [48,49]. Iron chelation therapy, such as deferasirox, is also used for those who chronically receive blood transfusions and have a higher risk of iron overload [50,51]. Also, supplemental therapy can also be provided depending on the patient’s condition [52].

Additional therapeutic measures

VOC or pain crisis is a common complaint for which pain evaluation and initiation of analgesic therapy are needed. Analgesics like acetaminophen or non-steroidal anti-inflammatory drugs (NSAIDs) can be used when symptoms are mild. If, however, pain is moderate to severe, opioids with or without NSAIDs can be used. Patients who present with fever must be immediately evaluated to exclude life-threatening infections. Administration of broad-spectrum antibiotics should not be delayed. For ACS, antibiotic therapy, analgesics, oxygen, beta-agonist inhalations, spirometry, and transfusion are among the measures to be potentially undertaken. Supportive therapeutic measures, including fluid administration and oxygen supplementation, might be indicated for other complications like stroke and sequestration crisis [4].

## Materials and methods

Subjects

This is a retrospective cohort study to observe selected cases at King Fahd Hospital of the University (KFHU) and correlate their clinical presentations to the laboratory data with more emphasis on transfusion dependence and its subsequent sequelae from the period of October 1, 2020, to September 30, 2021. The study sample size is 220 patients/cases. IRB/ethics committee approval was obtained (approval#: UGS-2021-01-418).

Inclusion and exclusion criteria

Inclusion criteria included SCD and homozygous and beta-thalassemia major. Exclusion criteria included pregnancy, cancer, and other unrelated comorbidities, i.e., ischemic heart disease, autoimmune disorders, hepatitis, and acute peptic ulcer.

Materials

Patient’s relevant data were collected using the hospital information system (HIS) QuadraMed (Reston, VA). Patients were coded with specific ID numbers to keep them and their information confidential. Eventually, Excel sheets (Microsoft Corporation, Redmond, WA) were used to write down the needed data based upon the frame of our research to facilitate analyzing it when it is collected completely to assess the outcomes.

Data analysis procedure

Data were analyzed by IBM SPSS 26 (IBM Corp., Armonk, NY). All categorical variables like sex, age groups, nationality, blood group type, and clinical presentations were presented as frequencies and percentages while all quantitative variables like age, lab parameters, and number of blood transfusion units were presented as mean and standard deviation (SD). The chi-square test was used to compare the proportion between two categorical variables while an independent sample t-test was used to compare the mean number of blood transfusion units between complications and a paired sample t-test was used to compare the mean lab parameters between the first and last follow-up visits. Spearman rho correlation coefficient was calculated between lab parameters and clinical presentations. A p-value of less than or equal to 0.05 was considered significant.

Variables

Independent variables included sex, age, and blood group type. Dependent variables included clinical presentations, laboratory parameters, complications, blood transfusion, and medications. Controlled variables were nil.

## Results

**Figure 1:**
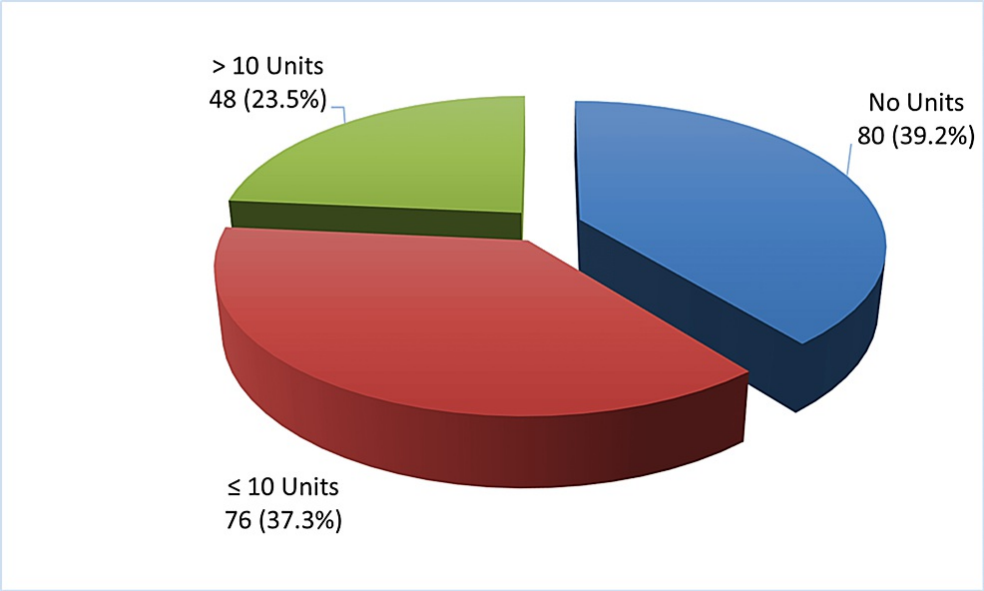
Blood transfusion in sickle cell disease cases (n = 204)

A total of 204 patients with SCD were included in this study. The mean age of the cases was 27.9 (±12.7) years. The majority of cases (108 patients who comprise 52.9% of the total cases of SCD) had an age between 11 and 30 years. Out of the 204 cases, 109 (53.4%) were males and 95 (46.6%) were females, and the male-to-female ratio was 1:0.87. Of the cases, 200 (98%) were Saudis while the remaining were non-Saudis of various nationalities. Out of the total, 116 (56.8%) cases were from Khobar, 31 (15.2%) were from Qatif, 23 (11.3%) were from Dammam, 21 (10.3%) were from Al-Ahsa, and eight (3.9%) were from Dhahran. Regarding blood type, 68 (33.3%) cases had blood type O+, followed by 40 (19.61%) cases of A+, and 24 (11.76%) cases of B+. Out of 204 cases, 80 (39.2%) cases received no blood transfusion along the course of their disease while 124 (60.8) cases have been transfused as follows: less than and equal to 10 units were transfused in 76 (37.3%) cases and >10 units were transfused in 48 (23.5%) cases. On average (±SD), 8.2 (15.2) units were transfused. Table 1 and Figure 1 show more details regarding the demographic data and transfusion units, respectively.

**Table 1:** Demographic characteristics of sickle cell disease cases (n = 204)

		Frequency	Percent
Age (years), mean (±SD) = 27.9 (±12.7)	<10	16	7.8
	11-30	108	52.9
	>30	80	39.2
Gender, M:F = 1:0.87	Male	109	53.4
	Female	95	46.6
Nationality	Saudi	200	98
	Non-Saudi	4	2
Region	Khobar	116	56.8
	Qatif	31	15.2
	Dammam	23	11.3
	Al-Ahsa	21	10.3
	Dhahran	8	3.9
	Others	5	2.5
Blood type	A+	40	19.61
	A-	2	0.98
	B+	24	11.76
	B-	2	0.98
	AB+	3	1.47
	O+	68	33.33
	O-	3	1.47

In βTM, however, a total of 16 cases were included in the study. The mean age of the cases was 27.4 (±14) years. Out of the 16 cases, eight (50%) had an age > 25 years while eight (50%) cases had an age ≤ 25 years. As far as sex is concerned, 10 (62.5%) were males and six (37.5%) were females, with a male-to-female ratio of 1:0.6. Fourteen (87.5%) cases had Saudi nationality while two patients were registered as non-Saudis. Out of the total, 13 (81.3%) cases were from Khobar, and the remaining three (18.7%) patients were from Dammam. Nine (56.25%) patients, which represent the majority of cases, had blood type O+, followed by two (12.5%) patients of B+, and A+ was seen in only one (6.25%) case of βTM. Out of the total cases, 14 (87.5%) have received blood transfusions. On average (SD), 149 (67) units were transfused. Refer to Table 2 for more details.

**Table 2:** Demographic characteristics of beta thalassemia major cases (n = 16)

		Frequency	Percent
Age (years), mean (SD) = 27.4 (14)	≤25	8	7.8
	>25	8	39.2
Gender	Male	10	62.5
	Female	6	37.5
Nationality	Saudi	14	87.5
	Non-Saudi	2	12.5
Region	Khobar	13	81.3
	Dammam	3	18.8
Blood type	A+	1	6.25
	B+	2	12.5
	AB+	1	6.25
	O+	9	56.25
	O-	1	6.25

Regarding SCD, a comparison between the clinical presentation of the first and last follow-up visits is presented in Table 3. Proportions of clinical presentations were high at the last follow-up visits as compared to the first visits. Back pain was reported by 23 (11.3%) cases at the last visit (p = 0.018), which was significantly high. A remarkably higher number of cases had generalized pain (14, 6.9%), abdominal pain (11, 5.4%), and palpitations at the last follow-up visits (6, 2.9%) (p-values = 0.016, 0.004, and 0.014, respectively). All other p-values were statistically insignificant.

**Table 3:** Clinical presentation of sickle cell disease cases (first visit vs. last visit) * P-values were calculated by the chi-square test. VOC = vaso-occlusive crisis; SOB = shortness of breath; RUQ = right upper quadrant.

Clinical presentation	Total	First visit	Last visit	P-values*
Back pain	33 (16.2%)	10 (4.9%)	23 (11.3%)	0.018
Leg pain	21 (10.3%)	8 (3.9%)	13 (6.4%)	0.25
Chest pain	18 (8.8%)	5 (2.5%)	13 (6.4%)	0.056
Generalized pain	18 (8.8%)	4 (2%)	14 (6.9%)	0.016
VOC	17 (8.3%)	8 (3.9%)	9 (4.4%)	0.8
Abdominal pain	12 (5.9%)	1 (0.5%)	11 (5.4%)	0.004
SOB	11 (5.4%)	5 (2.5%)	6 (2.9%)	0.8
Fever	10 (4.9%)	3 (1.5%)	7 (3.4%)	0.21
Arm pain	9 (4.4%)	5 (2.5%)	4 (2%)	0.73
Hip pain	8 (3.9%)	3 (1.5%)	5 (2.5%)	0.47
Knee pain	6 (2.9%)	3 (1.5%)	3 (1.5%)	1
Dizziness	6 (2.9%)	1 (0.5%)	5 (2.5%)	0.09
Palpitations	6 (2.9%)	0 (0%)	6 (2.9%)	0.014
Fatigue	6 (2.9%)	1 (0.5%)	5 (2.5%)	0.09
Headache	6 (2.9%)	2 (1%)	4 (2%)	0.406
RUQ pain	5 (2.5%)	3 (1.5%)	2 (1%)	0.64
Pallor	5 (2.5%)	2 (1%)	3 (1.5%)	0.64
Others	69 (33.8%)	26 (12.7%)	43 (21.1%)	0.023

The correlation (Spearman rho) between laboratory parameters and the presence of clinical presentations in SCD patients is presented in Table 4. At the first visit, cases presented with clinical features had a significant positive correlation with mean corpuscular volume (MCV) (ρ = 0.194, p = 0.006), chloride (Cl) (ρ = 0.354, p = 0.037), and alkaline phosphatase (ALP) (ρ = 0.315, p = 0.037), while creatinine, potassium (K), total bilirubin, and lactate dehydrogenase (LDH) had positive but insignificant correlation with clinical presentation and all other laboratory parameters had negative and insignificant correlation with clinical presentations. At last visit, cases with clinical features upon presentation had significant positive correlation with WBC (ρ = 0.282, p < 0.001), ferritin levels (ρ = 0.441, p = 0.001), total bilirubin (ρ = 0.289, p < 0.001), direct bilirubin (ρ = 0.317, p = 0.001), ALP (ρ = 0.276, p = 0.05), and LDH (ρ = 0.281, p < 0.001). While cases with clinical presentation had a significant negative correlation with hemoglobin (Hb) (ρ = -0.199, p = 0.006), hematocrit (ρ = -0.208, p = 0.004), CO2 (ρ = -0.365, p = 0.021), total protein (ρ = -0.373, p = 0.009), and albumin (ρ = -0.413, p = 0.017). Cases with clinical presentations were also positive but insignificantly correlated with reticulocyte count (ρ = 0.134, p = 0.101), anion gap (ρ = 0.338, p = 0.08), serum glutamic oxaloacetic transaminase (SGOT), and serum glutamic pyruvic transaminase (SGPT) levels. Additionally, a comparison between laboratory parameters of the first and last follow-up visit is presented in Table 5. The mean ± SD of WBC count was significantly low 9.72 ± 5.39 in the last follow-up visit as compared to the first visit 12.23 ± 6.89 (p < 0.001). Also, the mean ± SD of RBC (3.43 ± 0.85), sodium (Na) (137.72 ± 2.3), anion gap (9.5 ± 2.02), ALP (100.14 ± 49.3), SGOT (42.87 ± 17.81), and SGPT (66.94 ± 83.18) were significantly low at last visit as compared to first visit (p < 0.05). While the mean ± SD of MCV (87.63 ± 13.46) and Cl (106.04 ± 2.35) was significantly high at the last follow-up visit as compared to the first visit (p < 0.0001 and p = 0.001, respectively). All other parameters were statistically similar at the first and last follow-up visits, which can implicate no correlation.

**Table 4:** Correlation between laboratory parameters and clinical presentation in sickle cell disease (n = 204) * Spearman's rho correlation coefficient. ** Significant correlation between labs and clinical presentations. WBC = white blood cell; RBC = red blood cell; Hb = hemoglobin; Hct = hematocrit; MCV = mean corpuscular volume; RC = reticulocyte count; BUN = blood urea nitrogen; TB = total bilirubin; DB = direct bilirubin; TP = total protein; ALP = alkaline phosphatase; SGOT = serum glutamic oxaloacetic transaminase; SGPT = serum glutamic pyruvic transaminase; LDH = lactate dehydrogenase; GGTP = gamma-glutamyl transpeptidase.

Labs at 1st visit	Clinical presentation at 1st visit (ρ*)	P-values	Labs at 2nd visit	Clinical presentation at 2nd visit (ρ*)	P-values
WBC	-0.073	0.298	WBC	0.282**	<0.001
RBC	-0.109	0.124	RBC	-0.12	0.103
Hb	-0.002	0.978	Hb	-0.199**	0.006
Hct	0.002	0.974	Hct	-0.208**	0.004
MCV	0.194**	0.006	MCV	-0.066	0.368
RC	-0.046	0.553	RC	0.134	0.101
BUN	-0.036	0.788	BUN	0.098	0.397
Creatinine	0.045	0.675	Creatinine	-0.012	0.914
Na	-0.098	0.533	Na	-0.108	0.532
K	0.12	0.437	K	0.064	0.731
Cl	0.354**	0.037	Cl	0.01	0.947
CO2	-0.23	0.101	CO2	-0.365**	0.021
Anion gap	-0.259	0.134	Anion gap	0.338	0.079
Ferritin	-0.172	0.433	Ferritin	0.441**	0.001
TB	0.122	0.161	TB	0.289**	<0.001
DB	0.08	0.47	DB	0.317**	0.001
TP	-0.145	0.309	TP	-0.373**	0.009
Albumin	-0.181	0.271	Albumin	-0.413**	0.017
ALP	0.315**	0.037	ALP	0.276	0.05
SGOT	-0.035	0.771	SGOT	0.176	0.111
SGPT	-0.042	0.802	SGPT	0.185	0.259
LDH	0.067	0.42	LDH	0.281**	<0.001
GGTP	0.032	0.823	GGTP	0.206	0.083

**Table 5:** Comparison between laboratory parameters in sickle cell disease (first visit vs. last visit) * By paired sample t-test. WBC = white blood cell; RBC = red blood cell; Hb = hemoglobin; Hct = hematocrit; MCV = mean corpuscular volume; RC = reticulocyte count; BUN = blood urea nitrogen; TB = total bilirubin; DB = direct bilirubin; TP = total protein; ALP = alkaline phosphatase; SGOT = serum glutamic oxaloacetic transaminase; SGPT = serum glutamic pyruvic transaminase; LDH = lactate dehydrogenase; GGTP = gamma-glutamyl transpeptidase.

Laboratory parameter	Mean (±SD)		P-values*
	First visit	Last visit	
WBC	12.23 ± 6.89	9.72 ± 5.39	<0.001
RBC	3.6 ± 0.92	3.43 ± 0.85	0.002
Hb	9.52 ± 2.11	9.61 ± 1.85	0.557
Hct	28.78 ± 6.45	29.43 ± 5.87	0.143
MCV	81.29 ± 12.31	87.63 ± 13.46	<0.001
RC	6.66 ± 4.45	7.02 ± 4.07	0.368
BUN	7.34 ± 3.1	7.84 ± 3.08	0.287
Creatinine	0.49 ± 0.36	0.73 ± 0.95	0.078
Na	138.96 ± 3.3	137.72 ± 2.3	0.049
K	4.38 ± 0.56	4.32 ± 0.46	0.655
Cl	103.86 ± 2.49	106.04 ± 2.35	0.001
CO2	23.52 ± 3.81	22.25 ± 2.95	0.073
Anion gap	12.04 ± 4.26	9.5 ± 2.02	0.004
Ferritin	283.75 ± 292.06	2240.03 ± 3821.95	0.145
TB	3.93 ± 3.67	3.29 ± 2.86	0.072
DB	2.13 ± 3.92	1.44 ± 3.28	0.267
TP	7.93 ± 0.77	7.92 ± 0.83	0.952
Albumin	4.14 ± 0.53	4.21 ± 0.51	0.557
ALP	176.86 ± 161.49	100.14 ± 49.3	0.023
SGOT	56.02 ± 25.62	42.87 ± 17.81	0.001
SGPT	59.09 ± 43.77	46.35 ± 41.88	0.167
LDH	412.33 ± 172.71	429.61 ± 169.59	0.361
GGTP	118.1 ± 148.46	66.94 ± 83.18	0.05

**Figure 2:**
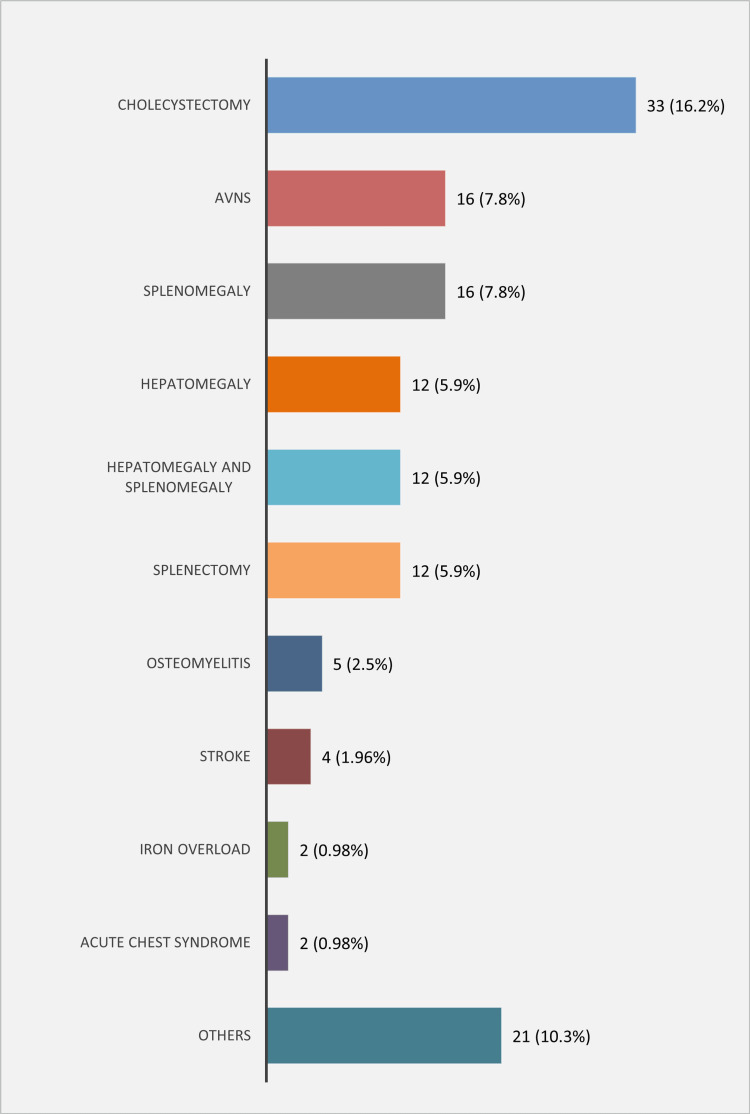
Complications of sickle cell disease (n = 204) AVNs = avascular necroses.

Figure 2 depicts the complications of cases with SCD. Cholecystectomy was the most frequent complication found in 33 (16.2%) cases. The aforementioned outcome could probably have been done secondary to cholecystitis or cholelithiasis as both are common in this disease and may necessitate such a measure to be undertaken. It is followed by AVN (16, 7.8%), splenomegaly (16, 7.8%), hepatomegaly (12, 5.6%), hepatosplenomegaly (12, 5.9%), splenectomy (12, 5.9%), osteomyelitis (5, 2.5%), and stroke (4, 1.96%), while ACS and iron overload were the two least frequent complications. It should be noted that splenectomy might have been indicated as in cases with severe splenomegaly.

A comparison between blood units given and complications in SCD cases is presented in Table 6. Cases with iron overload had significantly higher mean units of blood (83.5 units) as compared to cases without iron overload (7.4 units) (p < 0.0001). In other words, there is a strong correlation between frequent blood transfusion and iron overload. Similarly, cases with stroke had significantly higher mean units of blood (24 units) as compared to cases without stroke (7.9 units) (p = 0.035); the strong correlation between blood transfusion and stroke could be attributed to the management of stroke or secondary stroke prevention [53,54]. Cases with hepatomegaly had significantly higher mean units of blood (21.2 units) as compared to cases without hepatomegaly (7.9 units) (p = 0.005). Hepatomegaly could be caused by hemosiderosis secondary to blood transfusion and SCD itself rather than other etiologies [55,56]. Cases with osteomyelitis had significantly higher mean units of blood (44 units) as compared to cases without osteomyelitis (7.1 units) (p < 0.0001). The difference between the two means of all complications mentioned above is statistically significant. The difference between the two means of the rest of the complications was similar (p < 0.05) and statically insignificant.

**Table 6:** Comparison between blood units transfused and complications in sickle cell disease (n = 204) * By independent sample t-test.

Complications		Blood units		P-values*
		Mean	SD	
Iron overload	Yes	83.5	24.7	<0.0001
	No	7.4	13.2	
Cholecystectomy	Yes	7.9	9.5	0.9
	No	8.2	16.1	
Acute chest syndrome	Yes	22.5	23.3	0.181
	No	8	15.1	
Splenectomy	Yes	6.8	12.1	0.7
	No	8.3	15.4	
Stroke	Yes	24	29.4	0.035
	No	7.9	14.8	
Avascular necrosis	Yes	9.1	9.6	0.55
	No	7.1	13.4	
Osteomyelitis	Yes	44	40.5	<0.0001
	No	7.1	13.4	
Hepatomegaly	Yes	21.2	13.9	0.005
	No	7.9	15.9	
Splenomegaly	Yes	3.2	4.2	0.245
	No	7.9	15.9	
Hepatomegaly and splenomegaly	Yes	6.1	7.8	0.701
	No	7.9	15.9	

**Figure 3:**
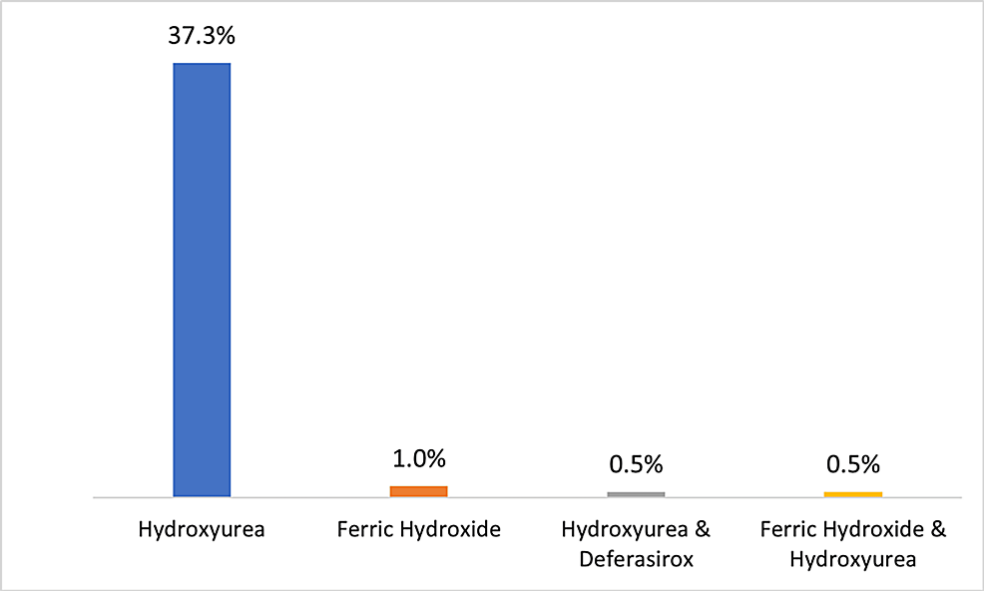
Medications used in sickle cell disease (n = 204)

Figure 3 shows the distribution of medications used by SCD patients. As can be noted, hydroxyurea was the most frequent medicine, which was prescribed to 37.3% of cases followed by ferric hydroxide, which was prescribed to only 1% of the total cases. A comparison between the mean Hb and MCV values of SCD cases using medications vs. those not on medications is presented in Table 7. Mean (±SD) Hb was statistically similar in the two situations at both first and last visits (p > 0.05) while mean MCV at the last follow-up visit was significantly high in cases with medications (91 ± 14) as compared to cases not on medications (85.2 ± 12.5) (p = 0.004).

**Table 7:** Comparison between mean Hb and MCV values in sickle cell disease cases on medications vs. not on medications * By independent sample t-test. Hb = hemoglobin; MCV = mean corpuscular volume.

Labs	Visits	On medication	Not on medication	P-values
Hb	First visit	9.4 ± 2.1	9.7 ± 2.1	0.25
	Last visit	9.8 ± 1.7	9.5 ± 1.9	0.31
MCV	First visit	82.5 ± 11.9	81.7 ± 12.7	0.65
	Last visit	91 ± 14	85.2 ± 12.5	0.004

The clinical presentation of βTM is presented in Table 8. Out of the total cases, two (12.5%) patients had no specific presentation except for low Hb as their main finding, two (12.5%) patients presented with abdominal pain, two (12.5%) presented with pallor, while fatigue, palpitation, itchiness, headache, shortness of breath (SOB), frothy urine, jaundice, and lower limb pain were each seen in one (6.25%) case. A comparison of laboratory parameters (first vs. last follow-up visits) is presented in Table 9. The mean ± SD of WBC count was significantly higher (26.4 ± 24.3) in the first visit as compared to the last follow-up visit (15.2 ± 9.3) (p = 0.031), while the mean ± SD of Hct (25.9 ± 3.4) was significantly high at last follow-up visit in comparison to the first visit (21.8 ± 6.3) (p = 0.023), and the mean ± SD of MCV (80 ± 9.8) was significantly higher at last follow-up visit than the first visit (69.9 ± 8.7) (p = 0.001). All other parameters were statistically similar at the first and last follow-up visits, which in turn shows no remarkable correlation.

**Table 8:** Clinical presentation of beta thalassemia major (n = 16)

Presentation	Number of cases	Percentages
Low hemoglobin	2	12.5
Abdominal pain	2	12.5
Pallor	2	12.5
Fatigue	1	6.25
Palpitation	1	6.25
Itching	1	6.25
Headache	1	6.25
Shortness of breath	1	6.25
Frothy urine	1	6.25
Jaundice	1	6.25
Lower limb pain	1	6.25

**Table 9:** Comparison between laboratory parameters in beta thalassemia major (first visit vs. last visit) (n = 16) WBC = white blood cell; RBC = red blood cell; MCV = mean corpuscular volume; RC = reticulocyte count; BUN = blood urea nitrogen; SGOT = serum glutamic oxaloacetic transaminase; SGPT = serum glutamic pyruvic transaminase; LDH = lactate dehydrogenase; GGTP = gamma-glutamyl transpeptidase.

Laboratory parameter	First visit	Last visit	P-values
WBC	26.4 ± 24.3	15.2 ± 9.3	0.031
RBC	18.1 ± 59.5	3.3 ± 0.9	0.336
Hemoglobin	7.3 ± 2	8.3 ± 1.1	0.059
Hematocrit	21.8 ± 6.3	25.9 ± 3.4	0.023
MCV	69.9 ± 8.7	80 ± 9.8	0.001
RC	3 ± 2.1	6.3 ± 1.9	0.121
BUN	13.5 ± 0.7	67 ± 70.7	0.475
Creatinine	0.5 ± 0.2	2.2 ± 2.8	0.388
Na	135.5 ± 3.5	138.5 ± 0.7	0.374
K	3.9 ± 0.3	3.8 ± 0.3	-
Cl	100 ± 2.8	104 ± 4.2	0.57
CO2	28.5 ± 0.7	20.5 ± 9.2	0.41
Anion gap	7 ± 0	14 ± 4.2	0.258
Ferritin	4218.8 ± 6847	5652.8 ± 5325.4	0.419
Total bilirubin	1.5 ± 0.4	1.5 ± 1.4	0.957
Direct bilirubin	0.3 ± 0.1	0.2 ± 0.1	0.478
Total protein	8.2 ± 0.8	7.7 ± 0.6	0.242
Albumin	3.5 ± 0.6	3.5 ± 0.9	1
Alkaline phosphatase	128.3 ± 49.1	114.7 ± 60.6	0.708
SGOT	76 ± 58	29.3 ± 11.6	0.225
SGPT	49 ± 35.9	29.7 ± 15.8	0.283
LDH	376 ± 432.1	193 ± 55.1	0.333
GGTP	46.4 ± 48	-	

**Figure 4:**
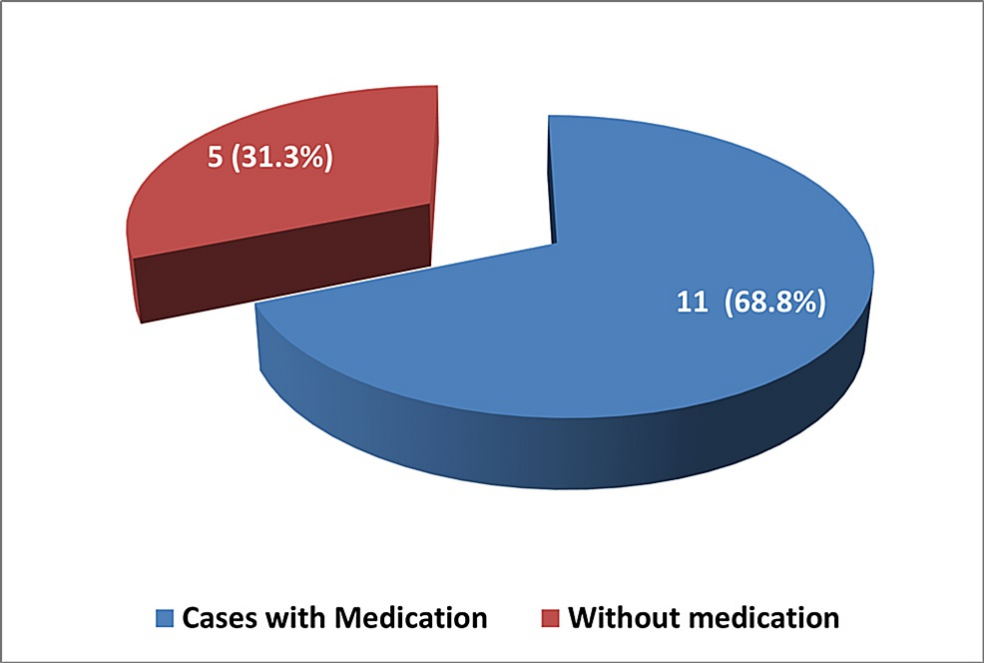
Medication (deferasirox) used in beta thalassemia major (n = 16)

Figure 4 depicts the distribution of medication in βTM patients who attended the hospital. Deferasirox, which is the only used medication, was prescribed to 11 (68.8%) cases while five (31.3%) cases were not using it or any other medication. Another comparison is presented in Table 10, which compares blood units given to patients with complications. Patients who underwent splenectomy, as mentioned before, could probably be attributed to severe splenomegaly and had significantly higher mean units of blood of 192 units as compared to cases who never had their spleen removed (129.3 units) (p = 0.023). While mean blood units were statistically similar in all other complications.

**Table 10:** Comparison between blood units transfused and complications in beta thalassemia major (n = 16) * By independent sample t-test.

		Blood units, mean ± SD	P-values
Iron overload	Yes	167 ± 24.04	0.71
	No	146.71 ± 72.21	
Hepatomegaly	Yes	181.33 ± 20.62	0.15
	No	130 ± 79.63	
Hypothyroidism	Yes	190.5 ± 57.28	0.376
	No	143.36 ± 69.01	
Cholecystectomy	Yes	166 ± 2.83	0.723
	No	146.86 ± 72.55	
Splenectomy	Yes	192.2 ± 30.42	0.029
	No	129.73 ± 72.09	

**Figure 5:**
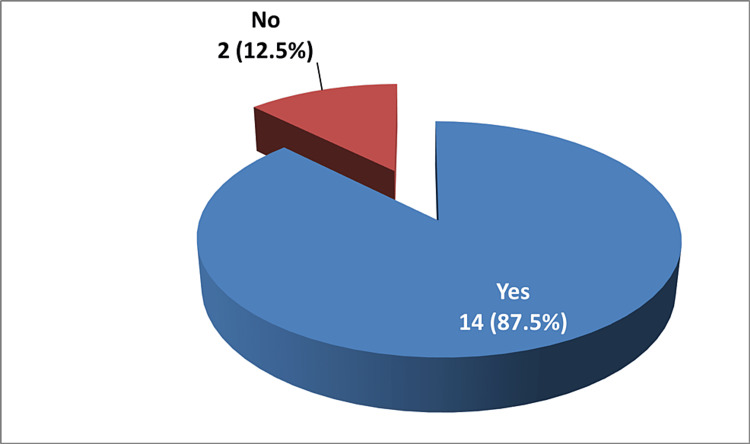
Blood transfusion in beta thalassemia major (n = 16)

In our retrospective study, out of the 16 βTM patients, 14 have been transfused, with a sum of 2388 blood units given overall. Refer to Figure 5 for further details. It is worth mentioning that the other two patients, who have no records of receiving blood transfusion at our hospital, are most likely getting their transfusion sessions at other hospitals or centers.

Concerning our study, a comparison between mean Hb and MCV values of cases using medication vs. those not on any medication in βTM is presented in Table 11. At the last follow-up visit, the mean ± SD of Hb was significantly higher in cases using deferasirox (9.36 ± 0.96) as compared to cases not using any medication (p = 0.008), while at the first visit, the mean Hb was statistically similar between the two groups. The mean ± SD of MCV was statistically similar in both cases at either visit (p > 0.05).

**Table 11:** Comparison between mean Hb and MCV values in beta thalassemia major cases on medications vs. not on medications (n = 16) * By independent sample t-test. Hb = hemoglobin; MCV = mean corpuscular volume.

		On medication	Not on medication	P-values
Hb	First visit	7.96 ± 2.42	7 ± 1.87	0.397
	Last visit	9.36 ± 0.96	7.87 ± 0.86	0.008
MCV	First visit	68.46 ± 13.22	70.53 ± 6.46	0.674
	Last visit	73.94 ± 13.32	82.77 ± 6.8	0.095

## Discussion

A retrospective analysis was reviewed, which included adult patients aged > 16 years at King’s College Hospital, London, and was concerned with the pattern of transfusion over a 10-year period (2000-2009). Out of 659 SCD patients, only 261 required transfusions at least once, with a sum of 8171 units being transfused. Percentages of blood units given to patients, who developed acute disease-related complications, have increased where the majority of transfusion therapy was related to VOC episodes and ACS, the rest is for acute stroke, and in one for cholecystitis/sickle cell hepatopathy/abdominal crisis/acute renal failure [57]. Another retrospective analysis was also reviewed, which included a descriptive study of children aged ≤ 16 years at Al-Sadaqa Teaching Hospital in Aden, Yemen, which was concerned with SCD patients who received a blood transfusion during hospital admissions over a one-year period (January-December 2014). Out of 217 SCD patients, only 169 cases required transfusions, with a sum of 275 RBC transfusion therapy. The main indications for transfusion were an anemic crisis, VOC, ACS, and stroke [58].

In another retrospective analysis, which included 158 patients from all age groups at the Hereditary Blood Disease Center, Al-Ahsa, Saudi Arabia, 65% of the cases were adults and 35% were pediatric patients. The analysis was concerned with blood demand and challenges for patients with βTM in the Eastern Province of the Kingdom over a three-year period (2017-2019). All 158 patients included in the analysis required chronic transfusion, with a sum of 14,508 units of PRBCs ordered and 9530 units were transfused. Acute hemolytic reactions, severe allergic reactions, and septic shock are some of the rapid-onset complications of chronic transfusion. Whereas delayed complications include transfusion-transmitted infections, delayed hemolytic transfusion reactions, and alloimmunizations [59].

Another retrospective study reported by the CDC included 407 patients from thalassemia centers located in Boston, Chicago, Philadelphia, New York, Oakland, Los Angeles, and Atlanta. The study was concerned with transfusion-related complications in thalassemia patients over a seven-year period (2004-2011). Out of 407 βTM patients, 327 required chronic transfusion, while only 38 were intermittently transfused, and 42 were never transfused, with an average total number of 149 units of blood. Some transfusion-related complications were hemosiderosis, which occurred in all groups, cardiac disease, gonadal failure, and hypothyroidism [60].

## Conclusions

Based on the aims of our study, different aspects of correlation have been found regarding the clinical picture, laboratory findings, and transfusion therapy and its associated complications in various patients of SCD and βTM, as our results and the subsequent discussion stated earlier. Firstly, when it comes to SCD, findings conclude that the majority of the patients show the homozygous pattern of the disease with the remaining, however, having the heterozygous form. Back pain, generalized pain, abdominal pain, and palpitations were, respectively, the most reported symptoms by patients. Different laboratory parameters were significant in patients who presented with symptoms on their first and later visits, including high levels of WBC, LDH, and ferritin. The latter can probably be associated with iron build-up with subsequent hemosiderosis in patients who are dependent on transfusion. The mean WBC was significantly low at later visits as compared to the initial one, which can be related to patients presenting with infections upon the initial course of the disease. Some of the patients were found to have undergone cholecystectomy before, which can be secondary to gallstone and/or cholecystitis, as either can be seen along the disease course. Acute chest syndrome and iron overload were the two least frequently encountered complications in those patients. As far as blood transfusion is concerned, patients with iron overload had a significantly higher mean unit of blood units in comparison to patients without iron overload, which shows that patients who are frequently transfused are more likely to develop iron overload as a transfusion sequela. Similarly, cases with stroke, hepatomegaly, and osteomyelitis were more significantly seen in patients with a higher frequency of transfusion. Medications-wise, hydroxyurea was the most frequently prescribed medicine for such patients.

As far as βTM patients are concerned, a few cases presented no specific clinical picture. They were only found to have low hemoglobin levels. Other patients, however, reported abdominal pain and pallor among other non-specific systemic features. For such patients, the mean WBC was higher at the first visits as compared to the last visits, while mean hematocrit and MCV levels were higher at the last visits as compared to the initial ones. Only those three markers were statistically significant. Deferasirox, for iron chelation, was the only medicine used by thalassemic patients. Patients who underwent splenectomy had significantly higher mean units of blood as compared to cases who never had their spleen removed. However, the mean blood units were statistically similar in all other complications.
